# Influence of Sampling Effort and Taxonomic Resolution on Benthic Macroinvertebrate Taxa Richness and Bioassessment in a Non-Wadable Hard-Bottom River (China)

**DOI:** 10.3390/biology14101444

**Published:** 2025-10-20

**Authors:** Jiaxuan Liu, Hongjia Shan, Chengxing Xia, Sen Ding

**Affiliations:** 1 State Key Laboratory of Environmental Criteria and Risk Assessment, Chinese Research Academy of Environmental Sciences, Beijing 100012, China; 2 College of Fisheries, Huazhong Agricultural University, Wuhan 430070, China; xiachengxing@mail.hzau.edu.cn

**Keywords:** biomonitoring, benthic macroinvertebrate, sampling strategy, biotic index

## Abstract

**Simple Summary:**

River health is usually assessed by monitoring benthic macroinvertebrate assemblage. However, it is a challenge to collect representative samples in non-wadable hard-bottom rivers. The purpose of this study is to determine how many sample replicates need to be collected in this river type in order to explore a reliable monitoring method of benthic macroinvertebrates. We chose a study reach of Danjiang river, China, which met the requirements of non-wadable hard substrates, and analyzed the changes in taxa richness at family and genus/species levels with different sample efforts. The results showed that the richness of benthic macroinvertebrates increased with the increase in sample replicates at both taxonomic levels. We found that six hand nets could provide an economical and stable assessment result of BMWP index. The coarser taxonomic resolution (i.e., family level) also makes the results more consistent and reduces the workload. This study initially provides a practical, economical, and reliable sampling strategy for monitoring the health status of similar rivers in China, and provides a reference for exploring bio-monitoring methods for other types of rivers.

**Abstract:**

Benthic macroinvertebrates are widely used for river ecosystem health monitoring, yet challenges remain in non-wadable rivers, particularly regarding sampling effort. We evaluated hand-net sampling efficiency at three sites along the Danjiang River (a Yangtze River tributary) by analyzing taxa richness across taxonomic levels under varying replicate numbers. In total, 61 taxa (41 families) of benthic macroinvertebrates were identified. Non-metric multidimensional scaling analysis indicated no significant spatiotemporal variation in community composition. However, sampling effort increased, and the benthic macroinvertebrate taxa richness at both genus/species and family levels also increased. At eight sample replicates, the taxa accumulation curve at the genus/species level did not show an asymptote, with the observed richness reaching 67–80% of the predicted values calculated by Jackknife 1. In contrast, the family-level curve exhibited a clear asymptotic trend, with the observed richness reaching 82–100% of the predicted values. As sampling effort increased, bias decreased and accuracy improved, particularly for family-level taxa. Additionally, the BMWP scores also increased with the sampling effort. When the replicate number was no less than six, the BMWP reached stable assessment grades for all cases. From the perspective of bioassessment in non-wadable rivers, the hand net is suitable for collecting benthic macroinvertebrates. However, there is a risk of underestimating taxa richness due to insufficient sampling effort. Using family-level taxa can partially mitigate the impacts caused by insufficient sampling efforts to a certain extent, but further validation is needed for other non-wadable rivers (e.g., those with soft substrates). In conclusion, our research results indicate that six replicate hand-net samplings in non-wadable hard-bottom rivers can be regarded as a cost-effective and reliable sampling method for benthic macroinvertebrate BMWP assessment. This strategy provides a relatively practical reference for the monitoring of benthic macroinvertebrate in the same type of rivers in China.

## 1. Introduction

Biological sampling optimization represents a critical and highly exploratory scientific challenge in ecological research [1,2,3]. This issue is profoundly influenced by biogeographic spatial distribution patterns and is closely tied to the specific objectives of biomonitoring programs. Designing effective biomonitoring programs for aquatic ecosystems presents greater complexities compared to terrestrial ecosystems [4], primarily due to our limited understanding of aquatic community dynamics. Additionally, the compositional distribution of aquatic communities often exhibits unpredictable fluctuations in response to various anthropogenic disturbances, including dam construction, water diversion, sewage discharge, and riparian zone development [5,6]. The main challenge in aquatic biomonitoring lies in achieving an optimal balance between cost investment and minimum sampling requirement for sample size, as practical constraints invariably preclude unlimited sampling possibilities [7].

Sampling effort in biomonitoring programs must be tailored to their specific objectives. For instance, river biodiversity monitoring, which aim to obtain a comprehensive species inventory, requires greater sampling effort to capture rare or endemic taxa, which are often missed due to their low abundance or restricted distribution [8,9]. In contrast, bioassessment monitoring focused on detecting environmental change or human disturbance can reduce requirements for sampling effort by relying on dominant biological groups to reflect the response of aquatic ecosystems to disturbances. This process often overlooks the negligible impact of rare taxa, ultimately achieving its goal of diagnosing ecological issues [10]. In biomonitoring, the negligible influence of rare taxa is often disregarded, since dominant groups are sufficient in diagnosing ecological issues. For instance, da Silva Santoset al. [11] relied on EPT taxa (i.e., Ephemeroptera, Plecoptera, and Trichoptera) to assess changes in river ecological conditions. Additionally, sampling effort is also related to habitat characteristics, as habitat complexity is directly correlated with species richness [12]. This means that habitats with complex structural types (e.g., riffles-pool sequences) require more sampling efforts to accurately represent their biological communities. Previous studies on sampling efforts have focused on sampling tools, time, and distance (or area) [2,7,10,13,14,15], as these are key to developing reasonable regional biological monitoring protocols.

Benthic macroinvertebrates are widely used to assess the health of aquatic ecosystems due to their ease of collection, limited mobility, and high sensitivity to environmental disturbances [16]. Numerous benthic macroinvertebrate assessment methods have been developed [17,18], but insufficient attention has been paid to the requirements for acquiring the data needed by each method. Theoretically, the sampling effort required for different assessment methods also depends on taxonomic resolution, finer taxonomic levels (e.g., species or genus) demand greater sampling intensity to capture detailed biodiversity information [10,19], whereas coarser levels (e.g., family or order) require less effort. Despite the widespread application of benthic macroinvertebrate assessment methods, both field monitoring and laboratory analysis involve significant time cost. Minimizing these time investments remains a practical scientific challenge in benthic macroinvertebrate monitoring.

The Biological Monitoring Working Party (BMWP) index is a widely used rapid bioassessment method for benthic macroinvertebrates [20,21,22]. Its advantage lies in requiring only family-level taxonomic data [23], but its evaluation results are influenced by the sampling efficiency of family-level taxa. Hand nets are commonly used for collecting benthic macroinvertebrates in non-wadable rivers in China, yet the sampling efficiency of this collection method remains unclear. The first aim of this study was to investigate the impact of the number of hand-net sample replicates on information acquisition at different taxonomic resolutions (family level vs. genus/species level). We empirically tested the hypothesis that a coarser taxonomic level (family) requires fewer replicates than a finer level (genus/species). The second aim was to calculate the BMWP index using family-level data, analyze how BMWP results vary with increasing replicate numbers, and establish the minimum sampling effort required to achieve stable evaluation results. By addressing these aims, we provide actionable insights for designing efficient and cost-effective river biomonitoring protocols that balance data reliability with field work investment.

## 2. Materials and Methods

### 2.1. Study Area

**Figure 1:**
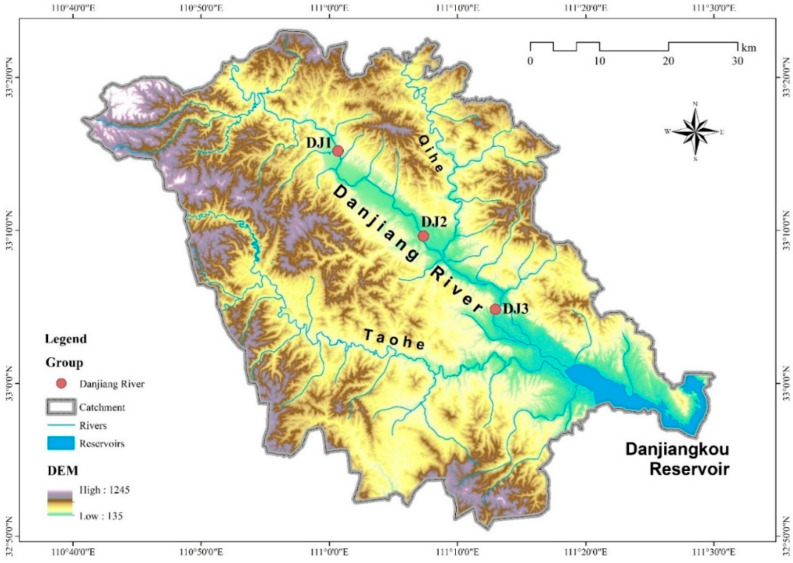
Location of sampling sites in the Danjiang River.

This study focused on a reach of the Danjiang River, a secondary tributary of the Yangtze River located in Henan Province. The river originates on the southern slopes of the Qinling Mountains within a subtropical monsoon semi-humid climate zone. The basin’s mean annual air temperature and precipitation range between 14.4 and 15.7 °C and 703.6–1173.4 mm, respectively. We established three sampling sites along a representative stretch between Jingziguan town and the Danjiangkou reservoir (Figure 1). This section of the river features a flat terrain and wide channel, with numerous densely vegetated islands scattered across the river. The riverbed is dominated by cobbles (with a coverage rate of over 90%). The riverbanks remain in a natural and stable state, and the riparian zone has high vegetation coverage, mainly consisting of shrubs. Meanwhile, this river section is less disturbed by human activities, thus maintaining good water quality conditions (Table S1).

### 2.2. Sample Collection and Identification

Benthic macroinvertebrates sampling was conducted during the pre-flood season (May–July) and post-flood season (September–October) in 2023. At each study site, we collected specimens using a D-frame hand net (mesh size = 0.5 mm, Wuhan Shuitiandi Technology Co., Ltd., Wuhan, China) and set up 8 transects along a single bank, with each transect being 5 m in length (Figure S1). The 5 m setup is based on the Chinese technical guideline for aquatic organisms [24]. The 8 transects are designed based on the workload of one day, including sample collection and on-site sorting of species. Sampling should primarily be confined to riffle areas and hard substrate regions, as these habitats are preferred for the hand-net method. Systematic collection was conducted from downstream to upstream to minimize disturbance effects. All field samples were immediately sieved through a 40-mesh screen to retain organisms while removing fine sediments. Benthic macroinvertebrates were hand-sorted from debris in a white porcelain pan. Each sample was separately preserved in 75% ethanol (Hebei Ruigui Medicine Technology Co., Ltd., Hengshui, China). Preserved specimens were sorted, counted, and identified to the lowest possible taxonomic level using a microscope (Olympus, Tokyo, Japan) and region taxonomic keys in a laboratory.

### 2.3. Sampling Effort Estimation

Species accumulation curves (SACs, known as sample-based rarefaction) were constructed using Mao Tau’s method [25] based on abundance data to illustrate changes in benthic macroinvertebrate taxa richness relative to sampling effort. To minimize random error, a randomization procedure with 100 permutations was applied [26], and the curves were smoothed by averaging 100 randomized iterations per sampling effort [27]. Obtaining all species within a region requires significant effort. Additionally, the basic data on the community composition of benthic macroinvertebrates in rivers are generally lacking. Therefore, in this study, the predicted taxa richness (i.e., theoretical true richness) at each site was estimated by the Jackknife 1 estimator [28], which showed better performance in bias and accuracy when estimating regional true richness [29]. All analyses were conducted in EstimateS 9.

### 2.4. Sampling Efficiency Evaluation

Precision and bias were used to evaluate the trend of errors in estimating the true richness of benthic macroinvertebrates across increasing sample size [30]. Precision means the repeatability of taxa richness obtained from independent transects and is estimated by the coefficient of variation (*CV*) and calculated as follows:

(1) $$CV=\frac{SD}{\bar{E}}$$

where *SD* is the standard deviation of the taxa richness of benthic macroinvertebrates and *Ē* is the average of 100 permutation estimates of taxa richness in each sample. *CV* < 20% is considered to have high precision [31] and a lower *CV* represents a higher precision.

Bias, also known as apparent error, refers to the difference between an individual measured value and the average measured value, and is used to measure the degree of deviation from the asymptote. Bias is measured by the scaled mean error (*SME*) as follows:

(2) $$SME=\frac{1}{An}\sum_{j=1}^{n}\left(E_{j}-A\right)$$

where *A* is the asymptote representing the theoretical true richness at each site, *E_j_* is the richness estimated for the *j*_th_ sample unit, and *n* is the number of replicates per sampling unit.

### 2.5. Biological Index Calculation

The river water quality was assessed by the BMWP index, which was calculated as follows:

(3) $$BMWP=\sum t_{i}$$

where *t_i_* is the environmental sensitivity value (ESV) of family *i*.

The score obtained in the BMWP index classifies water quality into five grades according to the Chinese adaptation system [24]: grade I (>145) classifies water quality as excellent, grade II (110–145) classifies water quality as good, grade III (73–109) classifies water quality as fair, grade IV (37–72) classifies water quality as bad, and grade V (<37) classifies water quality as poor.

### 2.6. Data Analysis

Non-metric multidimensional scaling (NMDS) was applied to visualize the similarities in benthic macroinvertebrate community structures across sampling seasons and sites. Complementarily, one-way analysis of similarities (ANOSIM) was performed with 999 permutations to assess spatial and temporal differences in community composition. An R value greater than zero from the ANOSIM analysis indicates that the within-group dissimilarities are smaller than the between-group dissimilarities, and higher R values mean more distinct between-group dissimilarities [31]. Both analyses were based on a Bray–Curtis similarity matrix derived from log(x + 1)-transformed abundance data. Specifically, NMDS was conducted using PC-ORD 5, while ANOSIM was implemented in PRIMER 7.

## 3. Results

A total of 61 taxa from 41 families of benthic macroinvertebrates were collected using a hand net over both seasons (Table 1). In the pre-flood season, 34 taxa (25 families) were identified, with site-level taxonomic richness ranging from 16 to 20. In contrast, the post-flood season yielded 54 taxa (40 families), with richness per site varying between 19 and 40.

**Figure 2:**
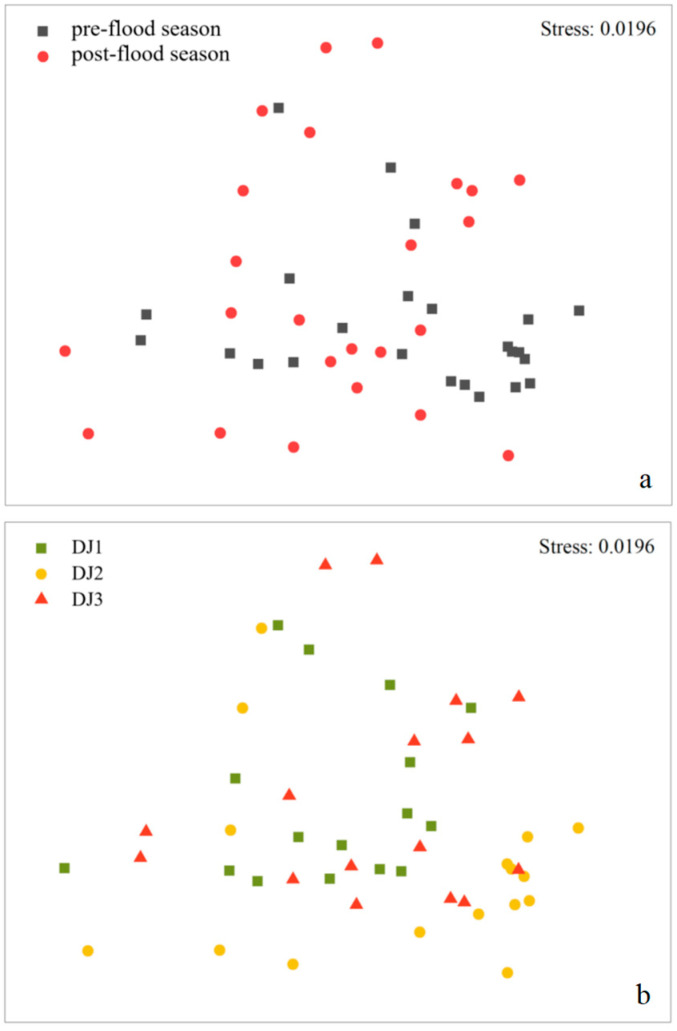
NMDS plot of benthic macroinvertebrate community composition between (**a**) seasons and (**b**) sites.

NMDS ordination revealed no distinct separation in benthic macroinvertebrate community composition between seasons (pre- vs. post-flood) or sites (DJ1, DJ2, DJ3) (Figure 2). This was supported by ANOSIM, which produced low separation values (R = 0.362 for seasons, *p* = 0.001 for seasons; R = 0.088~0.206 for sites, *p* = 0.001~0.002 for sites), further indicating minimal spatiotemporal variation in community structure.

**Figure 3:**
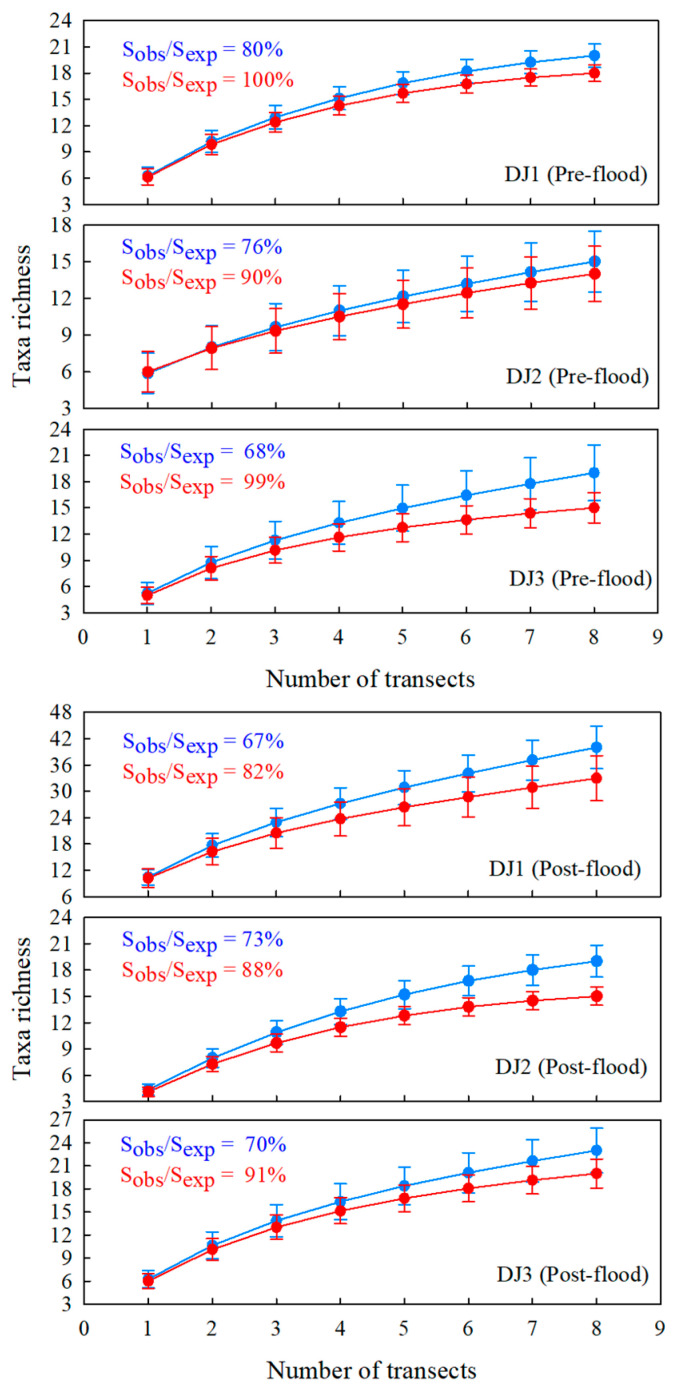
The taxa accumulation curves of benthic macroinvertebrates in the pre-flood season. Sobs means taxa richness observed. Sexp means taxa richness predicted by the Jackknife 1 estimator. Blue represents the results using the dataset of genus-species taxonomic level. Red represents the results using the dataset of family taxonomic level.

SACs at genus-species levels exhibited an asymptotic trend, where cumulative taxa richness increased progressively with sampling effort. However, none of the SACs across sites or seasons reached a stable asymptote (Figure 3), indicating that the current sampling intensity was insufficient in fully characterizing the potential richness of benthic macroinvertebrate community. Results from Jackknife 1 estimation showed that the observed taxa richness accounted for 67–80% of the predicted taxa richness at genus-species taxonomic resolutions. A similar pattern emerged at the family level, though with a closer approximation to complete sampling; while some curves approached an asymptote, none achieved full stabilization. Notably, observed family-level richness accounted for 82–100% of estimated total richness—substantially higher coverage than achieved for genus-species level identifications.

**Figure 4:**
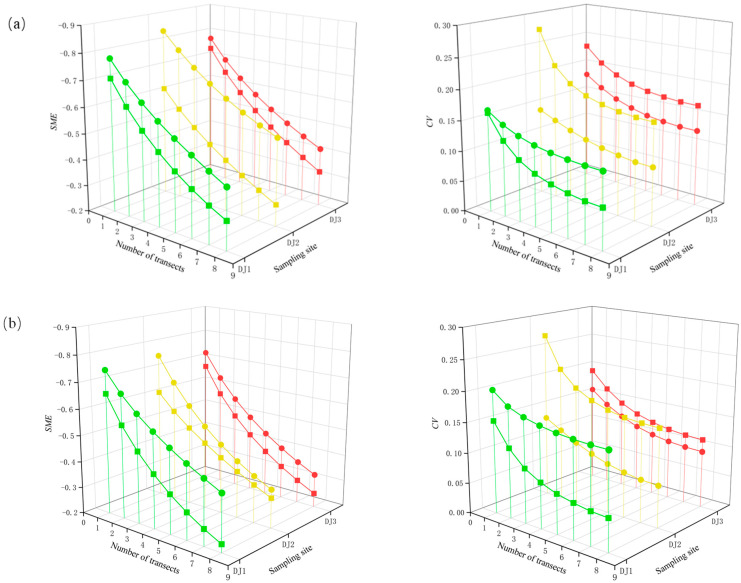
Changes in bias (scaled mean error, *SME*) and precision (coefficient of variation, *CV*) of benthic macroinvertebrate taxa richness estimates at (**a**) genus-species level and (**b**) family level in accordance with the sampling efforts. Square means pre-flood season. Circle means post-flood season. Green represents the result of DJI sampling site. Yellow represents the result of DJ2 sampling site. The red color represents the result of DJ3 sampling site.

At both the genus-species and family levels, *SME* and *CV* exhibited a negative response with increasing sampling effort (Figure 4). In terms of bias, all sites across two seasons showed a gradual decline in negative bias as sampling intensity increased, with no asymptotic trend observed at any taxonomic level. Conversely, the precision improved progressively, displaying a clear asymptotic trend when the number of sections exceeded six. Most *CV* values remained below 0.2, indicating consistently high precision. Seasonal comparisons revealed that the bias under varying sampling intensities was generally lower in the pre-flood season across all sites. However, while site DJ1 achieved higher precision in the pre-flood season, sites DJ2 and DJ3 exhibited greater precision in the post-flood season. Furthermore, compared to genus-species level, the family level demonstrated improved performance, with reduced bias and higher overall precision.

**Figure 5:**
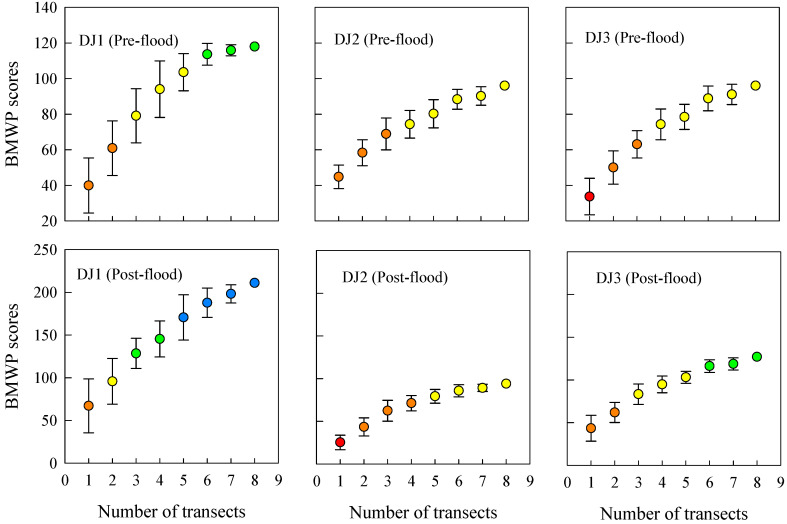
The assessment results of the Biological Monitoring Working Party (BMWP) index in accordance with the sampling effort. Circles represent the average score of the BMWP index obtained after 30 random permutations of the observed taxonomic composition. Bars represent the standard deviation of the BMWP index after 30 random permutations. Colors of circles represent assessment grade: blue (grade I); green (grade II); yellow (grade III); orange (grade IV); red (grade V).

With the increase in number of sampling replicates, the average BMWP index score across all sites and seasons gradually rose while the standard deviation progressively decreased (Figure 5). This pattern suggests that additional family-level taxa continue to be detected with enhanced sample effort, leading to more comprehensive and reliable ecological assessments. As the sampling effort increased, the BMWP index also demonstrated a change of 2–4 grades. The evaluation stabilized when the number of sampling replicates reached six, indicating that this level of effort provides a robust representation of the biological community. At this point, the average richness of family-level taxa ranged from 13 to 28, implying that the observed richness of family-level taxa accounted for 70.2% to 92.8% of their potential true richness (Figure 3). This finding underscores that six replicates strike an optimal balance between sampling efficiency and data reliability, ensuring accurate ecological assessments while minimizing unnecessary effort in this river.

## 4. Discussion

For non-wadable rivers with hard substrates, the available sampling tools include passive artificial samplers (e.g., Hester–Dendy sampler) and active hand nets. As a semi-quantitative sampling tool, hand nets can be applied not only in the hard substrate environment but also in soft substrate areas and aquatic vegetation-dense regions [32]. Given the specific conditions of the river section in this study, predominated by hard substrates, with a gentle riverbed slope yet relatively deep water, practical operations have verified that hand nets are a highly suitable sampling tool. According to the Chinese benthic macroinvertebrate monitoring protocol, the hand net is the recommended tool for such rivers, as they can effectively reduce the time cost input in routine monitoring work. In contrast, passive sampling methods require a higher investment of time [33]. However, a limitation of hand nets is their difficulty in application to extremely steep or deep riverbeds [31,34]. In this study, the sampling reach featured predominantly hard substrates, with a gentle riverbed slope rather than steep or deep sections. Thus, the use of a hand net was deemed highly appropriate for sampling under these conditions. In soft-substrate river systems, sediments are more evenly distributed and have a simpler structure. In such cases, mud collectors are more capable of accurately quantifying biodiversity. Hand nets, however, have an obvious advantage in such habitats. Sediment easily clogs the net mesh of hand-nets, and their use is accompanied by the collection of large amounts of detrital organic matter, which increases the time cost for subsequent cleaning and sorting of biological samples [7]. Surber samplers are applicable for sampling in some hard-substrate rivers, but in non-wadable rivers, they can only be used in shallow areas, resulting in relatively high constraints on their application.

The three non-wadable river sites in this study area exhibit diverse habitat types, including islands and alternating riffle-pool sequences. These complex habitats contribute to a highly random and spatially dispersed distribution pattern of benthic macroinvertebrates within the river channel. While our sampling protocol involved a continuous section along one riverbank, complete coverage of all habitat types could not be guaranteed. This methodological constraint introduced some degree of sampling variability, as evidenced by the presence of unique species at different sites (Figure S2). However, these site-unique species did not result in statistically significant differences in community composition (Figure 2). Although seasonal comparisons revealed no significant differences in community structure, we observed a notable increase in taxa richness in the post-flood season, especially at site DJ1 (Table 1). This pattern may be attributed to the geomorphic characteristics of the mountainous canyon in the study area’s upper reaches. High flow events induced by precipitation exert strong scouring effects, potentially displacing benthic macroinvertebrates and facilitating their passive downstream migration [35]. Furthermore, our analysis of spatial–temporal patterns showed that at higher taxonomic levels (family vs. genus-species), the number of unique macroinvertebrate taxa decreased significantly (Figure S2). As anticipated, this taxonomic aggregation led to a corresponding reduction in observed differences in community composition.

From the perspective of benthic macroinvertebrate taxa richness, the taxa accumulation curves at the genus-species level in this study did not exhibit a clear asymptotic trend (Figure 3). This suggests that additional sampling efforts would likely yield new taxa. Under the current maximum sampling intensity, the taxa collected across different sites accounted for only 67–80% of the estimated richness, with an average of 72%. Sampling strategies significantly influence the characterization of community structure [36]. For instance, Bady et al. found that ten replicate samples from a stream site captured only 50% of the potential benthic macroinvertebrate taxa richness [37]. Similarly, Ramos-Merchante and Prenda reported that ten hand-net replicates reflected approximately 70% of the potential taxa in a river site [30], while Bradey and Ormerod observed that five hand-net replicates could represent 70% of the taxa in highland streams [38]. These discrepancies may stem from variations in river habitat types. Habitat conditions strongly influence the collection of benthic macroinvertebrate taxa richness [39]. Furthermore, the selection of substrate samples from benthic mosaics introduces inherent randomness in capturing community structure information [40].

In biological monitoring and assessment programs, more detailed taxonomic levels can provide sufficient information for environment identification, while coarser taxonomic levels can reduce the consumption of funds and time [41]. As expected, the results demonstrated that the same sampling effort was more efficient for acquiring taxonomic information at the family level [28]. Silva et al. noted that family-level taxa accumulation curves stabilize earlier than those at the genus-species level [10]. In this study, family-level taxa accumulation curves approached an asymptote with increasing sampling effort, with particularly evident plateaus observed at site DJ3 in the pre-flood season and site DJ2 in the post-flood season (Figure 3). Furthermore, the recorded family-level richness accounted for 82–100% of the estimated potential richness, averaging 91.6%. De Bikuña et al. proposed that reducing the number of replicate samples (from 20 hand-net samples to just 5) could substantially lower the time investment while still retaining 64–83% of the total family-level richness [42]. The section-level accumulation curve provides a trade-off statistical method, which makes it clearer to determine which taxonomic level to use for advancing biological monitoring and assessment work.

From the perspective of bias and precision characteristics, the variation trends across different taxonomic resolutions are consistent, though differences exist among sampling sites. Overall, increasing sampling effort can reduce bias and enhance precision. The lower levels of bias are observed in the pre-flood season, which is likely due to the generally lower richness in this season. In terms of precision, most of sampling efforts achieve acceptable levels. Notably, CV of site D1 is lower in the pre-flood season, whereas site D2 and site D3 exhibit lower CV values in the post-flood season. These differences suggest that the substantial rise in taxa richness at site D1 in the post-flood season (Table 1) leads to greater variability in community composition among subsamples, resulting in a higher standard deviation. This may be caused by the immigration of a large number of new taxa during the flood season, which enhances the spatial patchiness of the community of benthic macroinvertebrate. Consequently, the representativeness of subsamples in reflecting the overall community composition decreases. This also explains why the overall precision in the pre-flood season was better than that in the post-flood season.

Uncertainty exists in the results of bioassessment, which depends on the collection of the total number of the actual taxa [30]. It follows that sampling effort is the key to guaranteeing reliable bioassessment results. In this study, the maximum sampling effort effectively captured family-level taxa richness. The BMWP index results, derived from family-level richness data, demonstrate that increased sampling effort enhances BMWP scores, with scores stabilizing at certain sites (i.e., site DJ1 in the pre-flood season, site DJ2 and site DJ3 in the post-flood season) under high sampling effort. Furthermore, in all cases, BMWP index assessment grade stabilizes after reaching six subsamples. Requirements for duplicate samples using the hand net are specified in the biomonitoring protocol of Sweden (5 replicates), France (8 replicates), and Portugal (6 replicates) [43]. In practical river biomonitoring work of China, 2 replicate samples are usually collected, and bioassessment relies on taxonomic data at the genus-species level, which obviously leads to an underestimation of evaluation results, even under the maximum sampling effort in this study. In contrast, using family-level taxonomic data (e.g., for the BMWP index) yields more reliable evaluation outcomes. From a cost-efficiency perspective, each additional replicate sample requires extra investment. However, based solely on the assessment results of the BMWP index, increasing the effort to six replicates ensures assessment stability and accuracy. This represents a tripling of field effort but yields a disproportionate gain in reliability, preventing the potentially far greater long-term costs associated with misdiagnosing a river’s ecological status.

Notably, previous studies have found that when the number of sample replicates is the same, a shorter sampling distance can also yield similar bioassessment results [14,44]. This study adopted a sampling distance of 5 m, and in terms of the sampling area per single sample, it is much larger than that required by other sampling protocols, such as the sampling requirements of AQEM [45]. In the future, the impact of shorter sampling distances on assessment results can be explored, with the expectation of further reducing sampling cost.

## 5. Conclusions

The effect of sampling effort on the biomonitoring and bioassessment has not been sufficiently emphasized and validated in China. This study found that in the Danjiang River, a non-wadable river with hard substrates, the taxonomic richness of benthic macroinvertebrate (including family level and genus/species level) increased with sampling effort, with an average of 90% of family-level taxonomic information obtained after eight sampling replicates. Compared to the family level, the genus/species level exhibited a greater inherent risk of underestimating taxonomic richness. The BMWP index score increased with sampling effort, and a sampling protocol with no fewer than 6 replicates ensured stable BMWP assessment results. From the perspective of optimizing future sampling efforts, further research could focus on refining sampling distances.

## Figures and Tables

**Table 1:** List of benthic macroinvertebrates and their relative abundance.

Order	Family (ESV)	Taxa	Pre-Flood Season			Post-Flood Season		
			DJ1	DJ2	DJ3	DJ1	DJ2	DJ3
Basommatophora	Lymnaeidae (3)	*Radix swinhoei*	++			++	+	+
	Physidae (3)	*Physa* sp.				+		+
	Planorbidae (3)	*Gyraulus convexiusculus*				+		
Mesogastropoda	Viviparidae (6)	*Bellamya* sp.				+		
	Stenothyridae (6)	*Stenothyra glabra*				+		+
	Semisulcospiridae (6)	*Semisulcospira cancellata*			+			
Veneroida	Corbiculidae (6)	*Corbicula* sp.	+		+	+		+
	Sphaeriidae (6)	*Sphaerium* sp.			+			+
Mytioida	Mytilidae (7)	*Limnoperna lacustris*	+++		++		+	+
Decapoda	Palaemonidae (6)	*Palaemon* sp.	++	+	+	+	+	+++
Hemiptera	Aphelocheiridae (10)	*Diplonychus* sp.						+
	Corixidae (5)	*Sigara* sp.				+	+	+
Coleoptera	Carabidae (5)	Carabidae sp.	+			++	+	+
	Chrysomelidae (5)	*Galerucella* sp.				+		
	Psephenidae (10)	Psephenidae sp.				+		
	Elmidae (8)	Elmidae sp.	++			+		
	Hydrophilidae (5)	*Enochrus* sp.			+			
	Dytiscidae (6)	*Rhantus* sp.	+	+	+	+		
		*Oreodytes* sp.			+		+	
Ephemeroptera	Heptageniidae (10)	*Heptagenia* sp.	++	+++	++	++		+
		*Ecdyonurus tobiironis*				++		
	Ephemerellidae (8)	*Serratella* sp.				+		+
		*Cincticostella* sp.				+		
	Polymitarcyidae (10)	*Ephoron* sp.		++		++	+	
	Caenidae (7)	*Caenis* sp.	+	++		++		
	Isonychiidae (8)	*Isonychia* sp.	+			+		
	Baetidae (7)	*Baetis* sp.	+	++	++		+	++
		*Cloeon* sp.		++		+		++
		*Baetiella* sp.					+	+
	Potamanthidae (10)	*Potamanthus* sp.		+++	++	++	++	+
Trichoptera	Phryganeidae (10)	Phryganeidae sp.						+
	Hydropsychidae (6)	Hydropsychidae sp.	+++	+++	++	++		+
	Polycentropodidae (7)	*Plectrocnemia* sp.						+
		*Polyplectropus* sp.				+		+
	Stenopsychidae (8)	*Stenopsyche* sp.	+	+	+			
	Rhyacophilidae (10)	*Rhyacophila* sp.		+		+	+	
Diptera	Tipulidae (8)	Tipulidae sp.	+	+		+		
		*Antocha bifida*	+			+		
		*Hexatoma* sp.	+					
		*Dicranota* sp.				+		
	Chironomidae (3)	*Chironomus riparus*		+				
		*Chironomus striatipennis*			+			
		*Natarsia* sp.		++	+		+	
		*Cricotopus* sp.	+					+
		*Hydrobaenus* sp.			++		+	
		*Robackia* sp.					+	
		*Saetheria* sp.			+			
		*Cryptochironomus* sp.					+	
		*Glyptotendipes* sp.				+		
		*Eukiefferiella claripennis*				+		
Odonata	Coenagrionidae (6)	*Platycnemis* sp.					+	
	Libellulidae (8)	*Sympetrum* sp.				+		
	Gomphidae (8)	Gomphidae sp.	++			+		
		*Lamelligomphus formosanus*				+		
Plecoptera	Perlidae (10)	*Paragnetina* sp.				+		
Megaloptera	Corydalidae (8)	*Protohermes* sp.	+		+	++	+	
Arhynchobdellida	Erpobdellidae (3)	*Herpobdella octoculata*				++		
Rhynchobdellida	Glossiphoniidae (3)	*Glossiphonia lata*		+		+		
Tubificida	Tubificidae (1)	*Limnodrilus* sp.	++	+	+	++	+	+
		*Branchiura sowerbyi*				+		
Tricladida	Dugesiidae (5)	*Dugesia* sp.				+		

Note: + represents the number of individuals fewer than 10. ++ represents the number of individuals fewer than 100. +++ represents the number of individuals more than 100.

## Data Availability

The data presented in this study are available on request from the corresponding author.

## Supplementary Materials

The following supporting information can be downloaded at: https://www.mdpi.com/article/10.3390/biology14101444/s1, Figure S1: Schematic diagram of sampling sections; Figure S2: The benthic macroinvertebrates richness in different seasons and sites at (a) genus-species taxonomic level and (b) family taxonomic level; Table S1: Characteristics of river channel morphological and water quality parameters (pre-/post-flood).
