# Supplementary material for: Influence of Sampling Effort and Taxonomic Resolution on Benthic Macroinvertebrate Taxa Richness and Bioassessment in a Non-Wadable Hard-Bottom River (China)
Source: Biology (Basel). 2025 Oct 20;14(10):1444. doi: 10.3390/biology14101444 (PMC12561569; doi:10.3390/biology14101444)
Supplement: Supplementary file 1 [file biology-14-01444-s001.zip › biology-3901439-supplementary.pdf]

Table S1. Characteristics of river channel morphological and water quality parameters (pre-/post-flood).

Site	Channel width (m)	Altitude (m)	pH	DO (mg/L)	Turbidity (NTU)	COD_{Mn} (mg/L)	BOD_5 (mg/L)	NH_3-N (mg/L)
DJ1	136.4	208	8.61 / 9.56	7.14 / 7.8	7.70 / 7.29	2.3 / 1.6	1.8 / 1.4	0.096 / 0.029
DJ2	159.6	190	8.48 / 9.05	7.37 / 7.62	9.34 / 13.8	2.6 / 2.3	2.8 / 1.4	0.072 / 0.097
DJ3	132.35	169	8.36 / 9.87	7.12 / 8.05	6.91 / 37.9	2.3 / 1.7	2.4 / 1.6	0.144 / 0.074

Note: DO, dissolved oxygen; COD_{Mn}, permanganate index; BOD_5, biochemical oxygen demand for 5 days; NH_3-N, ammonia nitrogen.

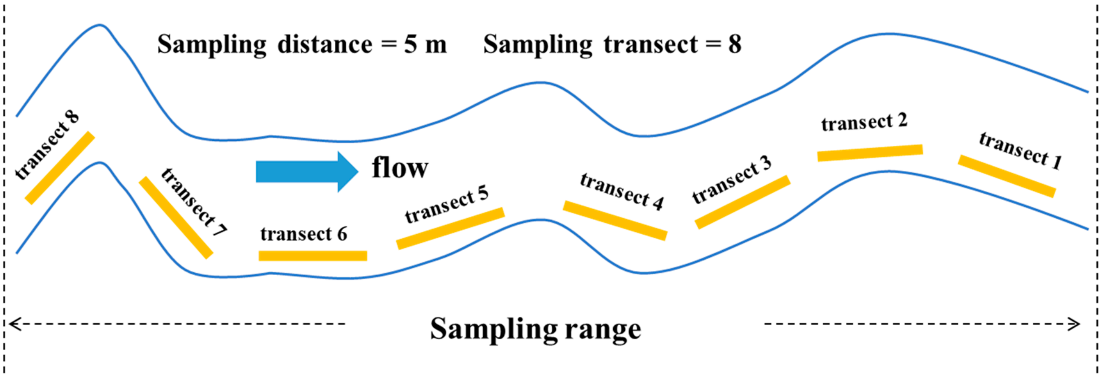

Figure S1. Schematic diagram of sampling sections.

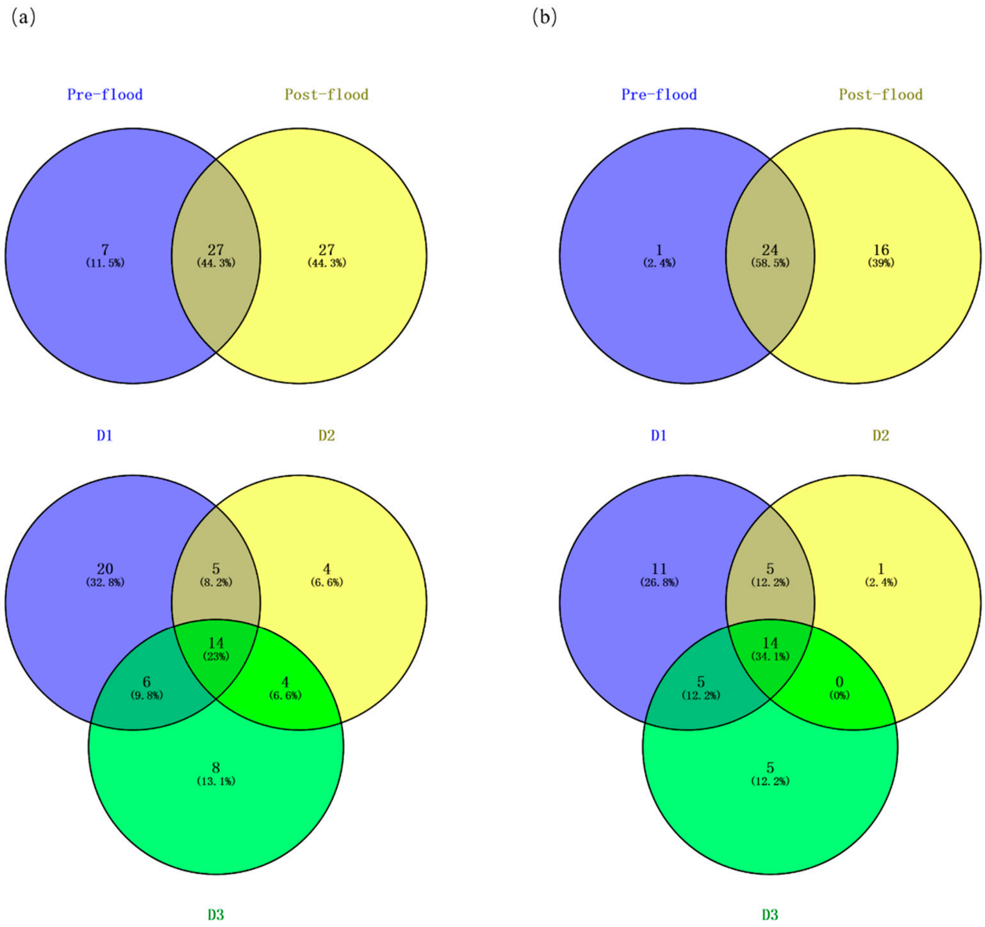

Figure S2. The benthic macroinvertebrates richness in different seasons and sites at (a) genus-species taxonomic level and (b) family taxonomic level.
